# Reversible Nonlinear I-V Behavior of ZnO-Decorated Graphene Nanoplatelets/Epoxy Resin Composites

**DOI:** 10.3390/polym12040951

**Published:** 2020-04-20

**Authors:** Yang Yuan, Zhaoming Qu, Qingguo Wang, Xiaoning Sun, Erwei Cheng

**Affiliations:** National Key Laboratory on Electromagnetic Environment Effects, Army Engineering University, Shijiazhuang 050003, China; spirit_yugi@163.com (Y.Y.); iamqzm3990@163.com (Z.Q.); sunxiaoning_8@163.com (X.S.); ew_cheng@163.com (E.C.)

**Keywords:** nonlinear I-V behavior, GNP-ZnO hybrids, reversibility, polymeric composites, switching threshold voltage

## Abstract

With the more serious threats from complex electromagnetic environments, composites composed of conductive or semiconductive fillers and polymeric matrices could exhibit excellent nonlinear I-V characteristics, and have drawn significant attention in the field of overvoltage protection. In this research, graphene nanoplatelets (GNPs) are decorated by ZnO and mixed into an epoxy resin (ER) matrix via solution blending to prepare composites. A characterization analysis and the I-V measurement results of the GNPs/ER composites indicate that ZnO nanoparticles are well bonded with GNPs and exhibit obvious nonlinear I-V behavior under proper applied voltage with high nonlinear coefficients. The switching threshold voltage and nonlinear coefficients could be controlled by adjusting the weight ratio of GNPs and ZnO of the filler. Moreover, compared with the poor recoverability of pure GNP-filled ER in previous research, the GNP-ZnO/ER composites exhibited excellent reversibility of nonlinear I-V behavior under multiple repetitive I-V measurements. And compared with different composites, the sample with a 1:8 weight ratio of GO to Zn(Ac)_2_ presents the smallest variation of switching threshold voltage at 158 V, with a standard deviation of 1.27% from among 20 measurements, which indicates the best reversibility. Finally, the conducting mechanism of the reversible nonlinear I-V characteristic is investigated and analyzed.

## 1. Introduction

To increase the safety and reliability of information equipment and integrated electronic systems, materials with obvious nonlinear I-V behavior—which should operate as insulators under normal working operation and transform to conductors rapidly when the external voltage surpasses a predetermined threshold [1,2,3]—have attracted significant attention and gradually begun to be used to protect electronic devices from electrostatic discharge and surge overvoltage, which could efficiently prolong the service life of devices [4].

In recent years, polymeric composites, which consist of a polymer matrix and conductive or semiconductive fillers, have exhibited nonlinear I-V characteristics and great utilization potential in the field of overvoltage protection because of their excellent properties [5,6,7,8,9,10,11]. In the report of S.I. White, silver nanowire-polystyrene nanocomposites were obtained, and their reversible conductive switching behavior was researched with increasing voltage at room temperature [12,13]. More and more related works have proved that, though the proper and effective reaction process, a mixture of an insulating polymer matrix and conductive fillers could possess nonlinear I-V behavior induced by applied voltage, making it useful for minimizing overvoltage damage.

Graphene has attracted extensive attention as an effective reinforcing material because of its excellent and distinctive properties [14,15,16], especially its superb electrical conductivity (6 × 10^5^ S/m [17,18,19]). In recent years, some researchers have reported that graphene nanoplatelets (GNPs) composed of few stacked layers can serve as a significant and potential filler which could improve the properties of polymeric composites [20,21,22,23,24,25,26,27]. In our previous investigation, a pure GNP-filled ER composites was prepared; it showed obvious nonlinear I-V behavior, but the poor reversibility prevented further application [28].

Hybrids, i.e., mixtures composed of two different nanoparticles, possess the properties of both components, and could be an effective strategy to enhance the properties of composites [29,30]. In the research of Yu et al., ZnO-decorated carbon nanotube (CNT) hybrids were obtained and used to prepare polymeric composites as fillers [31]; they showed reversible nonlinear I-V behavior and indicated that ZnO—a popular wide-gap semiconductor with many good characteristics in field-induced phase transition, especially reversibility—could be decorated on GNPs, effectively improving the poor reversibility of pure GNP-filled ER.

In this article, the GNPs were decorated with ZnO nanoparticles via the one-step solvothermal method, and GNP-ZnO/ER composites were obtained by solution blending. The characterization analysis and I-V measurement results of the GNP-ZnO/ER composites revealed excellent nonlinear I-V behavior with proper external voltage, and the switching threshold voltage was far below that of ZnO-based ceramic varistors. Moreover, the nonlinear I-V behavior of the GNP-ZnO/ER composites showed excellent and stable reversibility in multiple measurements, and the threshold voltage could be controlled by adjusting the weight ratio of GO to Zn(Ac)_2_ of the filler. Compared with previous research on pure GNP-filled ER, investigation of the conducting mechanism of the nonlinear I-V behavior verified that although the nonlinear coefficients of the pure GNP-filled ER are higher, the GNP-ZnO/ER composites are more practical for the actual application of overvoltage protection because of their excellent and reversible nonlinear I-V characteristics, which could reduce the risk of electronic equipment damage caused by reduplicative surge overvoltage while ensuring the normal operation.

The ZnO-Decorated graphene nanoplatelet hybrids and composites in our research were studied for their reversible nonlinear I-V characteristics and used for active overvoltage protection of electronic devices in complex electromagnetic environments. Although there have been some previous reports on GNPs/ZnO or GNP-ZnO hybrids and composites, they were almost all used as catalysts and photosensors. Moreover, compared to other hybrids or composites with nonlinear I-V characteristics, the GNP-ZnO hybrids and composites in our research exhibited excellent reversibility and stability. Furthermore, the methods for adjusting their nonlinear I-V characteristics were studied. Therefore, our research puts forward a relatively novel and practical way to improve the safety of electronic devices.

## 2. Materials and Methods

### 2.1. Materials

Graphene oxide (GO) was purchased from Tanfeng Tech Company (Suzhou, China) as the raw material of the GNPs. Zinc acetate (Zn(CH_3_COO)_2_·2H_2_O) and ethyl alcohol, purchased from Tianjin YongDa Chemical Reagent Company (Tianjin, China), were used as the raw material of ZnO and the main solvent. Epoxy resin (ER, E-51) was purchased from Chuzhou Hui-Sheng Electronic Material Company (Chuzhou, China) and selected as the insulating polymeric matrix. 2-Ethyl-4-methylimidazole (2E4MZ) was obtained from XiYa Reagent Company (analytical reagent, purity: 99%, Chengdu, China) and used as the curing agent of epoxy resin. Sodium hydroxide (NaOH), supplied by Tianjin DaLu Chemical Reagent Company (Tianjin, China), was used to maintain the pH of the reaction system. Hydrazine hydrate solution (analytical reagent, mass fraction: 85%) was purchased from Sinopharm Chemical Reagent Company, Tianjin, China) and used as the reductive agent to transform GO to GNPs.

### 2.2. Materials Preparation

In order to disperse the GO and Zn(CH_3_COO)_2_·2H_2_O solution, GO powders were first added to ethyl alcohol and ultrasonicated for 1 h. Then, Zn(CH_3_COO)_2_·2H_2_O particles were dissolved in the GO solution and ultrasonicated for 1 h. To maintain the pH at 10, NaOH solution was added dropwise. After stirring using a magnetic stirring apparatus for one hour, a small amount of hydrazine hydrate solution was added to the suspension and a RGO-Zn(OH)_2_ suspension was obtained after six hours of stirring at 90 °C. Then, the mixture was transferred into a Teflon-lined stainless steel autoclave and reacted at 180 °C for 20 h. After cooling, the reaction system was leached three times and the filter cake was dried in a vacuum freeze drier for 24 h to obtain the GNP-ZnO hybrid nanoparticles.

A mixture of GNP-ZnO hybrids and epoxy resin was obtained via solution blending with acetone. Then, the reaction system was stirred at 80 °C for several hours until the acetone totally evaporated. After that, a certain amount of 2E4MZ was added dropwise into the mixture and stirred for 1 min. Finally, the reactive system was vacuumized and transferred into a disposable dish to cure for 24 h at room temperature, and later, for 4 h at 100 °C to prepare the GNP-ZnO/ER composites.

### 2.3. Characterization and Measurements

Scanning electron microscopy (SEM, GeminiSEM 300 SEM instrument, Jena, Germany) and transmission electron microscopy (TEM, JEOL JEM-2100 TEM instrument, Tokyo, Japan) were used to analyze the microstructure and morphology of the GNP-ZnO hybrids and the fracture surface of the GNP-ZnO/ER composites. The crystal phase structure of the GNP-ZnO hybrids was characterized by X-ray diffraction (XRD, Beijing PuXi XD-6, Beijing, China). Fourier Transform IR (FTIR, Tianjin GangDong FTIR-650, Tianjin, China) was used to analyze the oxygen-containing groups and the reduction degree of the GNPs and GNP-ZnO hybrids. The Raman scattering characteristics of GNP-ZnO hybrids were detected by Raman spectroscopy (Raman, Horiba Scientific LabRAM HR Evolution, Tokyo, Japan). X-ray photoelectron spectroscopy (XPS, Thermo Scientific K-Alpha+, Massachusetts USA) was used to determine the atomic composition in the GNP-ZnO hybrids and the functional groups of carbon, oxygen, and zinc.

The I-V characteristics of the GNP-ZnO/ER composites were measured using a semiconductor parameter analyzer (Keithley 2600-PCT-4B, Keithley Instruments, Cleveland, USA), and a thin layer of silver conductive resin was pasted on both surfaces of the samples to ensure good contact with the test tools.

## 3. Results

### 3.1. Characterization of the GNP-ZnO Hybrids and Composites

**Figure 1:**
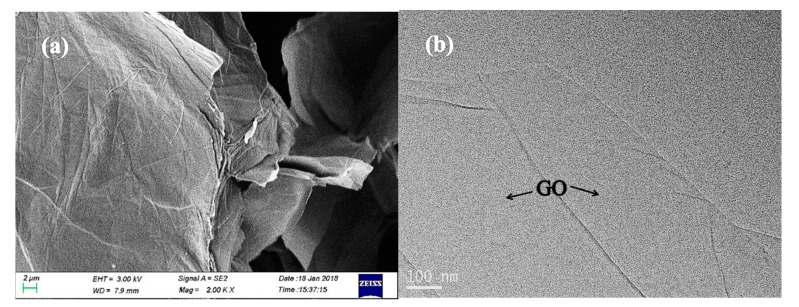
SEM micrograph (**a**) and TEM micrograph (**b**) of the GO powder.

Figure 1 shows SEM and TEM micrographs of the original GO powder. It can be seen that most of the GO flakes had good micromorphologies with no structural defects or stacking. As the raw material of GNPs, the GO selected in this research could provide surfaces with good morphological characteristics for the generation of ZnO, and could lay the foundation for follow-up reactions.

**Figure 2:**
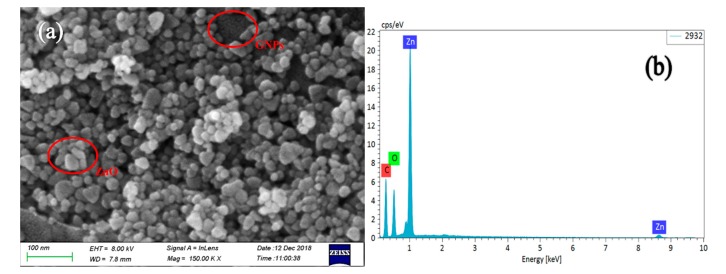
SEM micrograph (**a**) and EDS (**b**) of the GNP-ZnO hybrids powder.

Figure 2 presents the SEM micrographs and EDS measurement of the GNP-ZnO hybrids powder. From Figure 2a, it can be seen that many ZnO nanoparticles were evenly attached to the surface of the GNPs. Because of the attraction of positive and negative charges, the zinc ions (Zn^2+^) were attached to the oxygen-containing groups on the surface of GO, which could protect the surface morphology during the reduction process, so the majority of the GNP-ZnO hybrids flakes were composed of few stack layers and had very few defects and wrinkles.

Figure 2b shows the EDS measurement of the GNP-ZnO hybrid powder. Zn, C, and O were shown to be the main chemical elements on the films, which indicates that the nanoparticles on the film were probably the ZnO.

**Figure 3:**
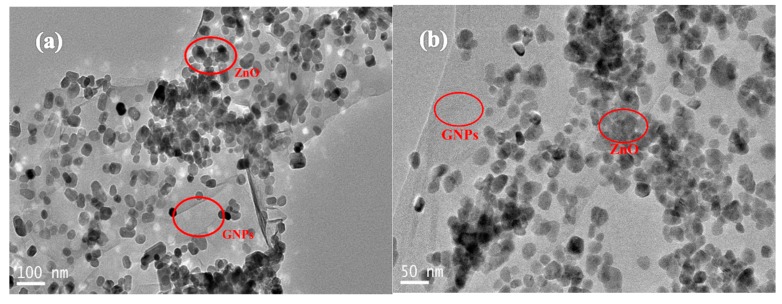
TEM micrograph (**a**) and highly-magnified TEM (**b**) of the GNP-ZnO hybrids suspension.

Figure 3 presents the TEM micrographs of the GNP-ZnO hybrids suspension. Although the reduction process could damage the initial structure of GO, the GNP flakes maintained high specific surface areas, and just a few defects, such as stacks and wrinkles, could be seen on the films because of the protection of Zn^2+^. Compared with Figure 1b, ZnO nanoparticles were distributed on the GNPs and there are no ZnO nanoparticles outside of the GNP flakes in Figure 3, as shown more clearly in the magnified micrograph in Figure 3b. Because ZnO nanoparticles were bonded to GNPs through oxygen-containing groups on the GO nanoplatelets which were mainly located at the edge of flakes and were not absolutely even, the ZnO nanoparticles were relatively concentrated at the edge of the GNPs, which is enough to keep closely arranged GNPs from coming into direct contact. So, in consideration of the purpose of the ZnO nanoparticle coating in this research, the nanoparticles in Figure 3 were strongly and evenly bonded to the GNPs surface and their size was in the range of 40−50 nm.

**Figure 4:**
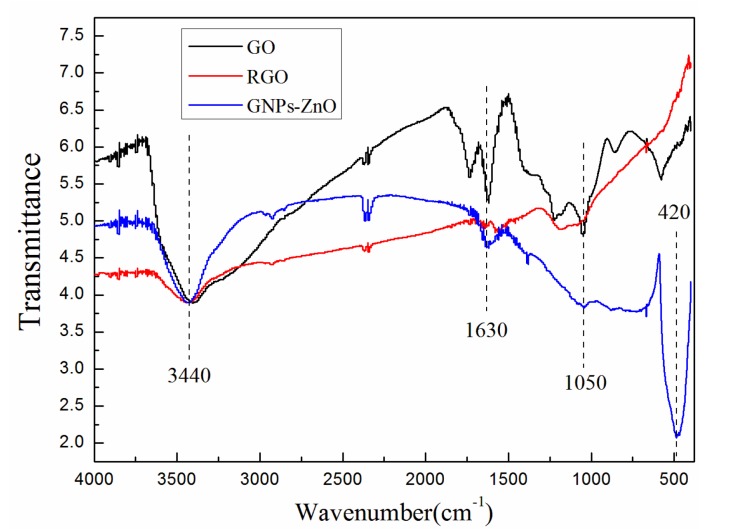
FTIR spectrum of GO, RGO, and GNP-ZnO hybrids.

During the reducing procedure from GO to GNPs, the reaction removed the oxygen-containing groups on the GO films and made the sp^3^-bonded structure transform into a sp^2^-bonded structure, which obviously increased the conductivity of the GNPs and cut the original GO flakes with large areas into smaller pieces [29,30]. Comparing Figure 2a with Figure 3, the area of GNP flakes decreased because of the reductive agent and high temperature during the reduction of GO and the synthesis of GNPs−ZnO hybrids, respectively. In order to analyze the reduction process of GNP-ZnO hybrids, the Fourier transform IR (FTIR) spectra of the GO, RGO, and GNP-ZnO hybrids were tested. As shown in Figure 4, the peaks of RGO and GNP-ZnO were obviously weaker than that of GO. Meanwhile, it could be seen that the peaks of GNP-ZnO, especially the O-H stretching vibration at 3440 cm^−1^, the C=O stretching vibration at 1630 cm^−1^, and the C-O-C stretching vibration at 1050 cm^−1^, were slightly stronger than those of RGO, indicating that there are still more oxygen-containing groups on GNP-ZnO than on RGO after the reaction procedure because the bonding of ZnO protected some groups from being removed. The obvious Zn-O stretching vibration at 420 cm^−1^ also showed that the ZnO nanoparticles had been obtained successfully and were strongly bonded to the GNPs surface.

**Figure 5:**
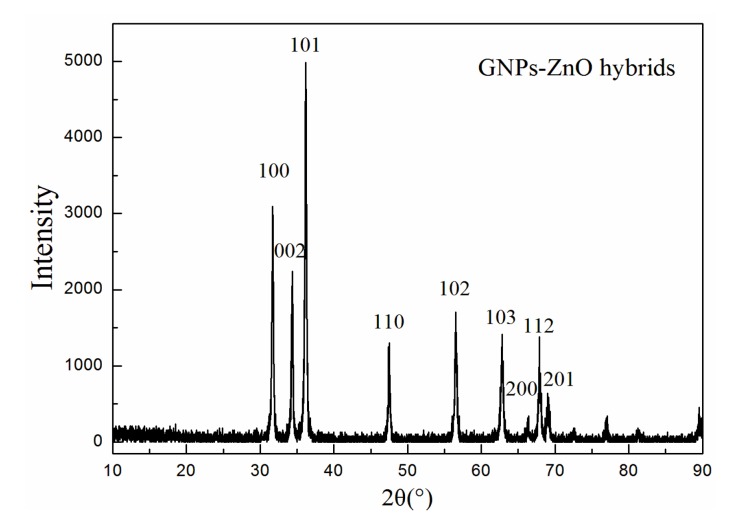
XRD spectrum of GO, RGO, and GNP-ZnO hybrids.

Figure 5 presents the XRD spectrum of the GNP-ZnO hybrids. According to the hexagonal structure (JCPDS No. 36-1451), the diffraction peaks at 31.6°, 34.2°, 36.1°, 47.3°, 56.5°, 62.8°, 66.3°, 67.8°, and 69.0° correspond to the crystal surfaces (100), (002), (101), (110), (102), (103), (200), (112), and (201) of ZnO, respectively, and there are no impurity peaks, indicating that the obtained ZnO had good hexagonal wurtzite structures and that the obtained hybrid nanoparticles were composed of well-coated hexagonal ZnO and GNPs.

**Figure 6:**
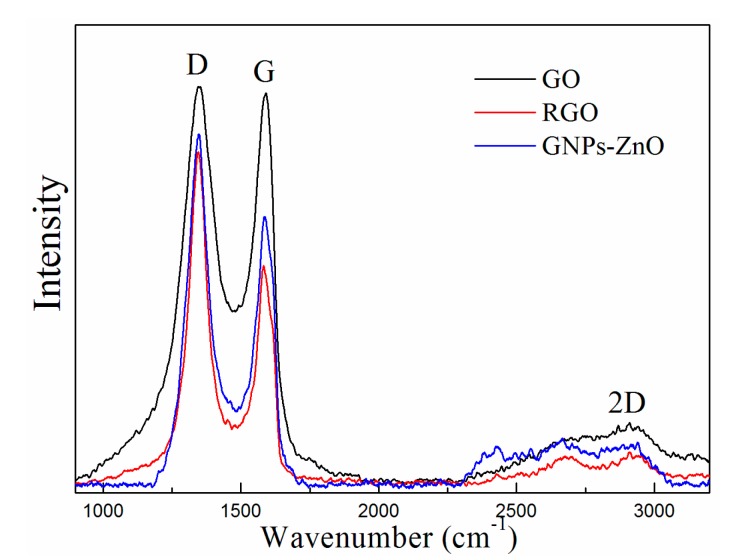
Raman spectrum of GNP-ZnO hybrids.

Figure 6 shows the Raman spectrum of the GO, RGO, and GNP-ZnO hybrids. The D-band (disordered band, at 1346 cm^−1^), G-band (sp^2^ carbon, at 1584 cm^−1^) and 2D-band (at 2675 cm^−1^) are presented in the figure. According to previous research, the D-band is caused by the structural defects in the sample, such as functional groups, sp^3^ bonds, and the small size of the crystalline domains. Respectively, the I_D_/I_G_ values of the GO, RGO, and GNP-ZnO hybrids were 1.016, 1.517, and 1.317, which indicates that the degree of disorder in the RGO and GNP-ZnO hybrids had increased because more defect-like holes having been created and the GNPs flakes having been cut into small pieces when the oxygen-containing groups on the GO were removed during the reduction. Moreover, the I_D_/I_G_ values of GNP-ZnO hybrids were less than those of RGO because of the coating, which indicated that the ZnO nanoparticles had successfully bonded to the surface of GNPs and could attenuate the defects of RGO.

**Figure 7:**
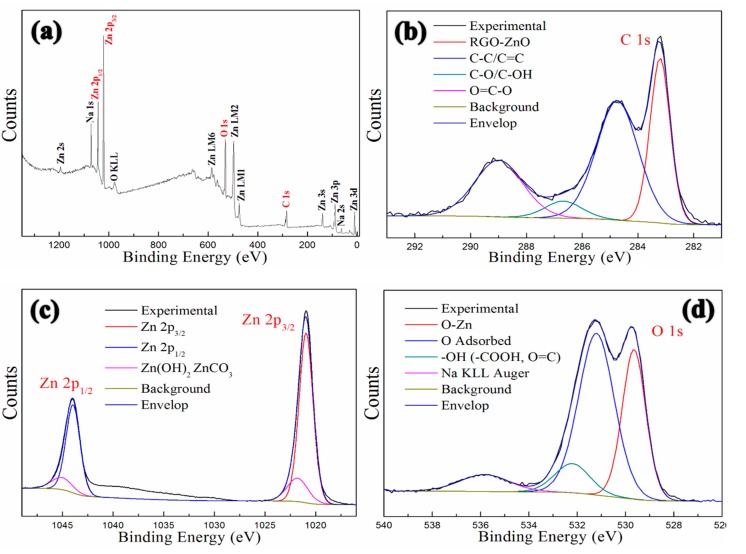
XPS spectra of GNP-ZnO hybrids: full spectrum (**a**), C 1s (**b**), Zn 2p (**c**), O 1s (**d**).

Figure 7 presents the XPS spectra of the GNP-ZnO hybrids. An XPS survey analysis of GNP-ZnO hybrids is shown in Figure 7a; it reveals several binding energy peaks, including carbon (C 1s, at 287.9 eV), zinc (Zn 2p_3/2_, at 1026.8 eV), zinc (Zn 2p_1/2_, at 1041.1 eV), oxygen (O 1s, at 539.2 eV), and so on. Figure 7b presents the XPS high resolution C 1s spectrum in the range of 281–290 eV; it shows that the binding energy peak C-C/C=C (at 284.8 eV) is much higher than the peaks C-O/C-OH (at 286.7 eV) and O=C-O (at 289.0 eV), indicating that most of the oxygen-containing groups on the GO were removed during the synthesis of GNP-ZnO hybrids. The binding energy peak RGO-ZnO at 283.4 eV, representing the bond between GNPs and ZnO, was very obvious, indicating that ZnO nanoparticles had been successfully and strongly bonded on the GNPs.

Figure 7c shows the XPS high resolution Zn 2p spectrum in the range of 1015–1050 eV, including Zn 2p_3/2_ and Zn 2p_1/2_ levels at 1021.0 eV and 1044.0 eV respectively. Meanwhile, Figure 7d presents the XPS high resolution O 1s spectrum in the range of 525–545 eV, in which the binding energy peak Zn-O at 529.7 eV is obvious. Combining the data in Figure 7c,d, it can be concluded that the nanoparticles on the surfaces of there GNPs were crystalline ZnO.

**Figure 8:**
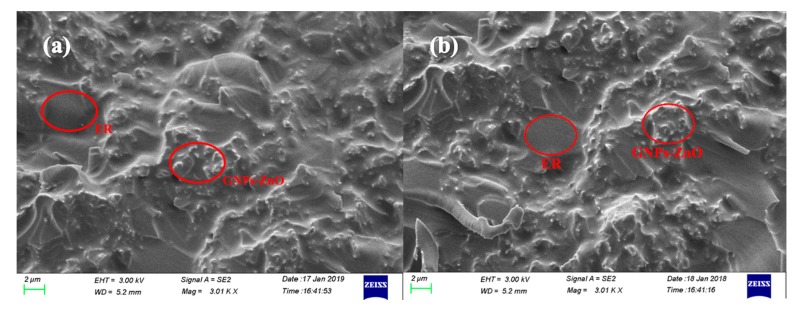
SEM micrographs of two different fracture surfaces (**a**) and (**b**) of the same GNP-ZnO/ER composites.

Figure 8 shows SEM micrographs of two different fracture surfaces of the same GNP-ZnO/ER composite sample. In Figure 8a,b, GNPs−ZnO hybrids are both well dispersed in ER matrix, while the agglomeration and the surfaces between GNPs−ZnO hybrids and ER matrix can barely be seen. Moreover, there was no obvious and substantial difference in the distributions of GNP-ZnO hybrids and the ER matrix between the two fracture surfaces, which indicates that the compatibility and dispersity of GNPs−ZnO hybrids were excellent.

### 3.2. Reversible Nonlinear I-V Characteristics of GNP-ZnO/ER Composites

To research the I-V characteristics of GNP-ZnO/ER composites, several reasonable weight ratios of GO to Zn(Ac)_2_, such as 1:20, 1:10, 1:8, 1:6.67, and 1:5, were chosen, and some specimens with a GNP-ZnO filler concentration of 20% were fabricated. There was a 30-s interval between the two I-V characteristics measurements.

**Figure 9:**
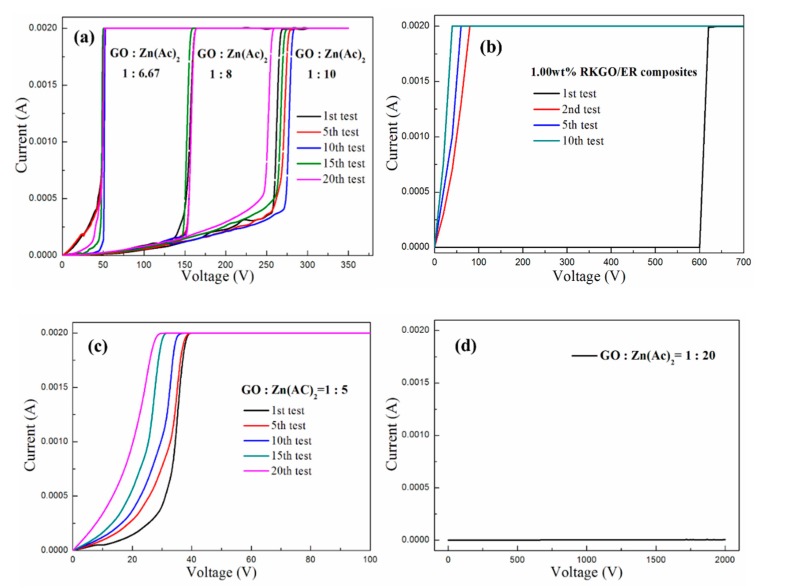
I-V characteristics of (**a**) the specimens with 1:10, 1:8, and 1:6.67 weight ratios of GO to Zn(Ac)_2_, (**b**) the specimen of pure GNPs/ER, (**c**) the specimens with 1:5 weight ratios of GO to Zn(Ac)_2_, and (**d**) the specimens with 1:20 weight ratios of GO to Zn(Ac)_2_.

Figure 9 presents the I-V characteristics of GNP-ZnO/ER composites with different weight ratios of GO to Zn(Ac)_2_. As shown in Figure 9a, the three GNP-ZnO/ER specimens with 1:10, 1:8, and 1:6.67 weight ratios of GO to Zn(Ac)_2_ all exhibited ohmic behavior at low voltage and obviously nonohmic behavior at high voltages. Additionally, with increasing the weight ratio of GO to Zn(Ac)_2_, the switching threshold voltage, as the boundary of linear region (Region 1) and nonlinear region (Region 2), decreased obviously. Moreover, compared with Figure 9b, which shows the measurement results of pure modified GNPs/ER (RKGO/ER) composites in our previous research, every GNP-ZnO/ER specimen in Figure 9a not only exhibited obvious nonlinear I-V behavior at different applied voltages, but also excellent reversibility for up to 20 measurements. The nonlinear I-V behavior of pure RKGO/ER composites only appeared once. Especially in Figure 9a, there are some slight deviations at the beginning of the measurements of the three specimens, because the thin and insulating epoxy matrix between ZnO was transformed to conductive paths under the applied voltage.

Meanwhile, two special cases are displayed in Figure 9c,d. Figure 9c shows the I-V characteristics of the GNP-ZnO/ER specimen with a 1:5 weight ratio of GO to Zn(Ac)_2_, which exhibited nonlinear I-V behavior at the beginning of the measurements, and the switching threshold voltage was lower than that of Figure 9a, but with further tests, the nonlinear I-V behavior of the specimen faded away and nearly disappeared after 20 measurements. The reason for this may have been the excessive GNPs in the specimen with the 1:5 weight ratio of GO to Zn(Ac)_2_, which was too much to be well coated by ZnO and caused poor reversibility, as with pure GNPs/ER. In contrast, because of the insufficient number of GNPs, the GNP-ZnO/ER specimen with a 1:20 weight ratio of GO to Zn(Ac)_2_ could not become conductive, even under an applied voltage of 2000 V.

The nonlinear coefficient *α* can be calculated by taking the ratio of log(*I*_2_/*I*_1_) to log(*V*_2_/*V*_1_), namely *α* = [log(*I*_2_) − log*(I*_1_)]/[log(*V*_2_) − log(*V*_1_)], where *I*_1_ and *I*_2_ are the measured current values under the voltages of *V*_1_ and *V*_2_, respectively[31,32,33,34,35]. Table 1 shows the average nonlinear coefficients of GNP-ZnO/ER specimens filled with various weight ratios of GO to Zn(Ac)_2_ with several tests, in which the coefficient *α* values of Region 1 are in the range of 1.34 to 2.99, and with the increasing weight ratios of GO to Zn(Ac)_2_, the *α* values decrease slightly. The coefficient *α* values of Region 2 are much bigger than those in Region 1, i.e., in the range of 22.01 to 86.74, with the same increasing trend as that of Region 1.

Table 2 presents the change range of switching threshold voltage and the standard deviation Δ of GNP-ZnO/ER specimens with 1:10, 1:8, and 1:6.67 weight ratios of GO to Zn(Ac)_2_. The result indicated that the specimen with the weight ratio of 1:8 has the smallest Δ. So, in combination with the curves of Figure 9a, the specimen with a 1:8 weight ratio of GO to Zn(Ac)_2_ was shown to possess the best reversibility.

In summary, the GNP-ZnO/ER composites with various weight ratios of GO to Zn(Ac)_2_ showed not only excellent nonlinear I-V behavior and controlled the switching threshold voltage and nonlinear coefficients by adjusting the weight ratio of GO to Zn(Ac)_2_, but also exhibited excellent reversibility under multiple tests compared with previous pure GNPs/ER composites, which could be more practical and feasible for the actual application of overvoltage protection. Moreover, in order to obtain GNP-ZnO/ER with good nonlinear I-V behavior and reversibility, the weight ratio of GO to Zn(Ac)_2_ should be set in the proper range, such as 1:10 to 1:6.67. The sample with a 1:8 weight ratio of GO to Zn(Ac)_2_ presented the smallest variation of switching threshold voltage at 158 V, with a standard deviation of 1.27%, indicating the best reversibility.

## 4. Discussion

In previous research, filler-matrix charge transfer, field-enhancing tunneling, electronic hopping, and filamentary conduction were studied to interpret the mechanism of the nonlinear I-V behavior of various types of inhomogeneous materials [36,37,38,39,40]. Because of the strong bond between the GNPs and ZnO nanoparticles, the equipotential model is constructed and the free electrons near the Fermi level of GNPs could hop to the ZnO under the applied voltage. Then, these electrons could transport to other adjacent ZnO directly or through the thin epoxy. Therefore, the conductive paths in GNP-ZnO/ER composites are composed of GNP-ZnO heterojunctions and ZnO−epoxy−ZnO units. According to the analysis above, the mechanism of the nonlinear I-V behavior of GNP-ZnO/ER composites could be interpreted by the indications given in Figure 8.

**Figure 10:**
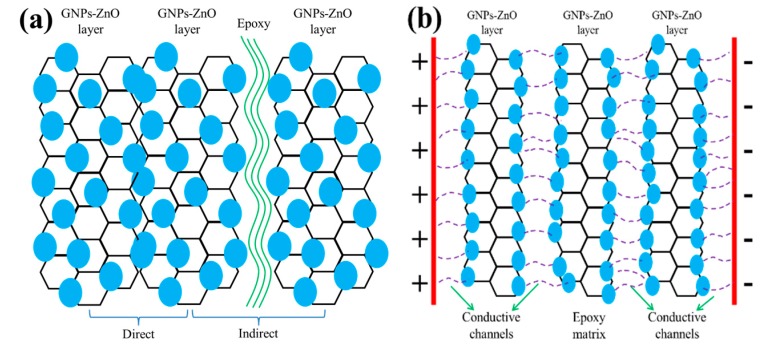
(**a**) Schematic of GNP-ZnO hybrids in composites, (**b**) illustration of the GNP-ZnO/ER structure under a high applied voltage sweep.

Figure 10a shows the microstructure model of the GNP-ZnO hybrids and epoxy resin in composites, in which GNPs play the most important role in conductive paths because of their good conductivity and extremely specific surface area; additionally, the ZnO nanoparticles evenly line up on the surface of GNPs, which is the key of reversibility. In Figure 10a, two different connection types between fillers were presented. Direct connection is the first type, in which the nanoparticles touch each other directly and form the GNP-ZnO–ZnO-GNPs unit. Because the band gap of ZnO is much wider than that of GNPs, this metal-semiconductor heterojunction could be thought of as a typical Schottky barrier between the interface of the GNPs and the ZnO, which provides the main condition for a quantum tunneling effect [41] and enables electrons to hop from GNPs to ZnO and form an electric current through migration and hopping under applied voltage. Therefore, the GNP-ZnO–ZnO-GNP unit could cause nonlinear I-V behavior. Another type is indirect connection, in which a sufficiently thin epoxy matrix exists between the GNP-ZnO hybrids and forms a ZnO-epoxy-ZnO unit. This structure is similar to the metal-insulator-metal model with a double-Schottky barrier-like direct connection [42,43]. According to previous reports on the double-Schottky barrier, the electrons with low or high energy levels in GNPs and ZnO pass through the barrier by quantum tunneling or hopping, respectively, which forms current I in the double-Schottky barrier and follows the relationship:

(1) $$I=I_{t}+I_{h}=\alpha V+\alpha \beta V^{3}+\frac{1}{R_{0}}V\cdot \exp\left(\frac{eV}{k_{0}T}\right),$$

where $I_{t}$ and $I_{h}$ are the current that forms by the tunneling and hopping effects, respectively, α and β are both constants, and V is the applied voltage. Thus, at relatively low voltages, the GNP-ZnO/ER composites continue to act as insulators. An electronic hopping and tunneling effect could occur between adjacent GNP-ZnO hybrids through a sufficiently thin insulating epoxy matrix layer once the applied voltage reaches the switching threshold voltage, which results in nonlinear I-V behavior of the GNP-ZnO/ER composites and some slight deviations during the early measurements. Above all, the nonlinear I-V behavior is caused by both the electron hopping and quantum tunneling occurring at the double-Schottky barrier of the GNP-ZnO–ZnO-GNP heterojunction and the ZnO-epoxy-ZnO unit.

Figure 10b presents an illustration of the GNP-ZnO/ER specimen under multiple applied voltage sweeps. With the proper filler concentration and weight ratios of GO to Zn(Ac)_2_, conductive paths are formed between adjacent ZnO nanoparticles because of the Joul heating of the epoxy matrix layer under a high applied voltage, which transforms the electron transport path from ZnO-epoxy-ZnO to GNP-ZnO–ZnO-GNP, i.e., a more stable and recoverable unit. However, in pure GNPs/ER composites, the GNPs-epoxy-GNPs unit transforms into a GNP-GNP unit, also by Joul heating, which is more unstable than the GNP-ZnO–ZnO-GNP unit and exhibits irreversible nonlinear I-V behavior, as shown in Figure 9b. As a consequence, GNP-ZnO/ER composites demonstrate not only obvious nonlinear I-V behavior, but also excellent reversibility.

## 5. Conclusions

The reversible nonlinear I-V behavior of the GNP-ZnO/ER composites was investigated. Compared with previous pure GNPs/ER composites, the GNP-ZnO/ER composites not only exhibited excellent nonlinear I-V behavior and high nonlinear coefficients under certain applied voltage, but also possessed stable reversibility with the proper weight ratio of GO to Zn(Ac)_2_. Therefore, the GNP-ZnO/ER composites obtained in this research are more practical and feasible for the protection of electronic devices, and could be a potential supplement of graphene and ZnO composites in the field of overvoltage protection. The electronic hopping and tunneling caused by GNP-ZnO–ZnO-GNPs heterojunctions and the double-Schottky barrier of ZnO-epoxy-ZnO units contributes to the reversible nonlinear I-V behavior of the GNP-ZnO/ER composites. Moreover, the switching threshold voltage and nonlinear coefficients can be controlled by adjusting the weight ratio of GO to Zn(Ac)_2_ of the filler; the sample with a 1:8 weight ratio of GO to Zn(Ac)_2_ presented the smallest variation of switching threshold voltage, i.e., 158 V, with a standard deviation of 1.27%, indicating the best reversibility.

## Figures and Tables

**Table 1:** Nonlinear coefficients of GNP-ZnO/ER specimens filled with various weight ratios of GO to Zn(Ac)_2_.

GNP-ZnO/ER Composites		
Weight Ratio	Region 1	Region 2
1:10	2.99	86.74
1:8	1.92	31.54
1:6.67	1.34	22.01

**Table 2:** Switching threshold voltage of GNP-ZnO/ER specimens filled with various weight ratios of GO to Zn(Ac)_2_.

GNP-ZnO/ER Composites		
Weight Ratio	Range (V)	Δ (%)
1:10	267.50 ± 12.50	4.67
1:8	158.00 ± 2.00	1.27
1:6.67	51.00 ± 1.00	1.96
